# Analyzing changes in the complexity of climate in the last four decades using MERRA-2 radiation data

**DOI:** 10.1038/s41598-020-57917-8

**Published:** 2020-01-22

**Authors:** Alfonso Delgado-Bonal, Alexander Marshak, Yuekui Yang, Daniel Holdaway

**Affiliations:** 1 NASA Goddard Space Flight Center, Earth Sciences Division, Greenbelt, Maryland United States; 2 Universities Space Research Association, Columbia, Maryland United States; 3 NASA Goddard Space Flight Center, Global Modeling and Assimilation Office, Greenbelt, MD United States; 4 University Corporation for Atmospheric Research, Boulder, Colorado United States

**Keywords:** Atmospheric science, Statistical physics, thermodynamics and nonlinear dynamics

## Abstract

The energy balance of the Earth is controlled by the shortwave and longwave radiation emitted to space. Changes in the thermodynamic state of the system over time affect climate and are noticeable when viewing the system as a whole. In this paper, we study the changes in the complexity of climate in the last four decades using data from the Modern-Era Retrospective analysis for Research and Applications, Version 2 (MERRA-2). First, we study the complexity of the shortwave and longwave radiation fields independently using Approximate Entropy and Sample Entropy, observing that the rate of complexity change is faster for shortwave radiation. Then, we study the causality of those changes using Transfer Entropy to capture the non-linear dynamics of climate, showing that the changes are mainly driven by the variations in shortwave radiation. The observed behavior of climatic complexity could be explained by the changes in cloud amount, and we research that possibility by investigating its evolution from a complexity perspective using data from the International Satellite Cloud Climatology Project (ISCCP).

## Introduction

Climate is a complex system^1^ composed of many different processes operating at all spatiotemporal scales, showing non-linear relations between its variables in long time spans^2^ or its acceleration in the last four decades^3^. Understanding the changes in these non-linear relations is of the utmost importance for understanding current climate change and its implications for the future^4,5^. Great efforts have been made in the last years to characterize those variations due to its importance for future abrupt changes in the structure of climate, being it potentially harmful to biodiversity and society^6,7^.

The atmosphere of the Earth can be considered as a closed non-linear thermodynamic system which basically only exchanges radiation with its environment^8^. The solar radiation flux maintains the atmosphere in a non-equilibrium thermodynamics situation, generating a climate dynamics which has an influence on the temperature of the planet. The energy balance is modulated by the incoming solar radiation and the outgoing shortwave and longwave Earth’s radiation. Changes in the energy balance of the atmosphere are the drivers of climate change.

Nowadays, it is believed that the Earth absorbs more energy from the Sun than it is emitted to the space, creating a situation of energy imbalance^9,10^. Although those differences can be monitored from the ground, the use of satellite sensors to provide measures of the fluxes of energy to and from Earth offers a more precise view of the system^11^. The evolution of the energy imbalance has been investigated using satellite data and models, and it seems to be increasing in the last decades^12,13^. While those changes have been argued to be due to human activities^14^, the questions of which of the parameters are changing faster and if that is affecting the dynamics of the system are still open.

When reaching the Earth, part of the incoming solar radiation is reflected off clouds and the surface as shortwave radiation (SW). Changes in cloud distribution or the surface albedo affect this flux and change the energy balance^15^. In the last four decades, changes in cloud distribution in low-level clouds such as subtropical stratocumulus have been of great importance^16^ since they have the ability to reflect large amounts of radiation back to space but do not reduce significantly the outgoing terrestrial radiation.

The rest of the radiation is absorbed by the atmosphere or passes through to the Earth’s surface, and the heat generated by this absorption is then emitted as longwave radiation (LW). Changes in the chemical composition of the atmosphere or the temperature of the oceans affect this flux in short and long term scales^17^. In general, perturbations in either shortwave or longwave radiation will lead to an energy imbalance which has a direct impact on the dynamics of the Earth system^18^.

The dynamics of climate is essential in forecasting climate change and its recent evolution can be analyzed by studying the complexity of the shortwave and longwave radiation fields. Several techniques have been developed to estimate the complexity of a time series, such as the Lyapunov exponent, Kolmogorov complexity, correlation dimension or Lempel-Ziv complexity. In this work, our interest lies in i) classifying the complexity and ii) studying the evolution of non-linear relations between shortwave and longwave radiation. We perform the first task by using two techniques based on Information Theory and Chaos Theory, namely Approximate Entropy (ApEn)^19^ and Sample Entropy (SampEn)^20^. Both algorithms are able to discern changing complexity and we apply them to the last four decades of MERRA-2 radiation data^21^.

ApEn and SampEn are a family of statistics to quantify randomness and complexity, useful to classify systems and applicable to stochastic and deterministic processes^22^. They look for repetitions of patterns in a dataset, quantifying the complexity of the data. Larger values of these statistics denote higher complexity and lower values imply organization and a higher degree of predictability. Those techniques are especially useful for characterizing non-linear systems, where other techniques are less effective on determining characteristics of the models^23^. The algorithms have been successfully applied in a wide range of disciplines, from physiological research^20,24^ to finance^25,26^ or Earth sciences^27,28^.

Shortwave and longwave radiation are strongly related and changes in one influence the behavior of the other. Thus, the questions of which one of those has had more influence on current climate changes, and how their relationship has evolved in the recent past, cannot be established by analyzing each time series separately. However, even when analyzing the series together, the usual measures of correlation are symmetric and do not give information about causality, i.e., which of the variables is driving the system.

To measure causality, a statistical concept called Granger causality was developed based on the idea of prediction^29^. The idea behind causality is that if a variable X1 “Granger-causes” another variable X2, then the prediction of X2 is more accurate including the unique information contained in X1 about X2 than considering X2 alone. If X1 and X2 are independent, then the inclusion of X1 does not help to predict X2. Granger causality leans on the idea that the cause occurs before the effect, and therefore it points those variables who drive the system.

Unlike correlation measures, Granger causality is an asymmetrical measure based on linear regression modeling of stochastic processes. However, as the relationship between shortwave and longwave radiation does not have to be linear, the use of vector autoregressive methods and the analysis of Granger causality would not provide any meaningful information^30^. A different approach to measure causality which has proved its efficiency is called Convergent Cross-Mapping (CCM)^31,32^. CCM is based on the theory of dynamical systems and its application is suitable for systems where causal variables have synergistic effects, expanding the scope Granger causality which is best suited for purely stochastic systems of linear relations.

Here, we keep our work within the Information Theory domain and present an analysis of causality based on Transfer Entropy^33^, another measure which allows us to determine the causality between two series of data without being restricted to an underlying linear dynamics. This technique, which has been applied to different fields from biochemistry^34^ to Earth^35^ and Space sciences^36^, allows us to capture the exchange of information between two systems and the directionality of the flux, determining the influence of one variable into the other. While autoregressive models are limited to linear relations, Transfer Entropy is a general technique which can be used to analyze any system, being equivalent to Granger causality for Gaussian variables^37^. In this work, we study the causality between shortwave and longwave radiation in a yearly basis since 1980 using Transfer Entropy and determine the flux and direction of the information exchange between the two data series and its trend.

## Data and Methods

### Data

The Modern-Era Retrospective analysis for Research and Applications, Version 2 (MERRA-2), is maintained by the NASA Global Modeling and Assimilation Office, providing data beginning in 1980 until the present. The project is an effort of assimilation of space-based observations and reanalysis of data to represent the climate system^21^, and has been used and validated to analyze important climatic features such as the atmospheric water balance^38^, aerosols^39,40^, precipitation^41^ or the radiation budget and cloud radiative effects^42^. MERRA-2 produces a regularly-gridded, homogeneous record of the global atmosphere, providing temporally consistent time series with high spatial and temporal (hourly) resolution, incorporating observations from recent satellite instruments.

**Figure 1:**
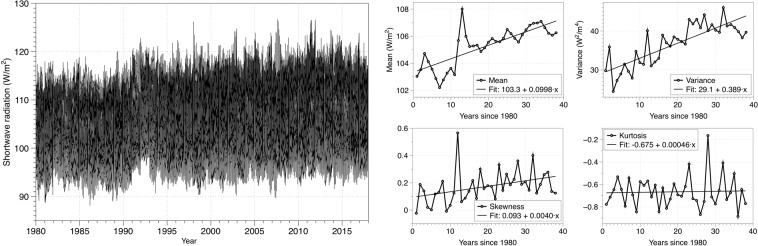
Shortwave radiation (left) and its usual statistical measures (right).

**Figure 2:**
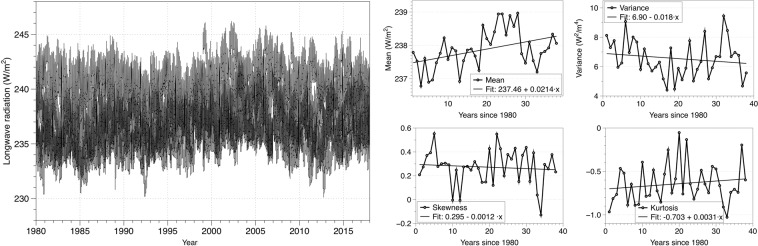
Longwave radiation (left) and its usual statistical measures (right).

Here we use the MERRA-2 hourly data record since 1980 for global shortwave and longwave radiation, analyzing each year (approximately 8760 points) of the time series separately. Before applying the complexity algorithms to the dataset, we transform the values using a log-ratio (log *s*_*t*_/*s*_*t*−1_) transformation to induce stationarity and to be able to measure the real complexity of the data, not any trends or seasonal cycles which may exist^24^. A table summarizing the main statistic values for both kinds of radiation is included in the Supplementary Material and plotted in Figs. 1 and 2. The dataset is freely accesible at the MERRA-2 project site.

The International Satellite Cloud Climatology Project (ISCCP) began in 1982 as part of the World Climate Research Programme (WCRP) and currently contains information about cloud distribution and properties since 1984. The project collects and analyzes satellite radiance measurements to improve understanding of climate research in general and the importance of clouds on the radiation balance in particular. The latest release is named “H,” and the version used in this research is the ISCCP-Basic data, containing instantaneous measures at 3 hours intervals with 1 deg equal angle^43^.

### Approximate entropy

A description of the algorithm can be found in^24^, and a comprehensive step by step tutorial with visual examples is available in^44^. Both Approximate Entropy and Sample Entropy require the selection of two parameters for their calculations, the embedding dimension *m* (the size of the template being compared) and the noise filter *r* (points within a distance of *r* are considered equal). The idea behind these complexity algorithms is to look for patterns within the data. If the patterns are repeated many times, the series is considered to be of low complexity and predictable; on the contrary, if it is difficult to find patterns, the series is considered highly complex and unpredictable. In this work we use an embedding dimension of *m* = 2, derived from a Mutual Information analysis^45^ (see Supplementary Material), and a tolerance of *r* = 0.1 σ, a value close to the maximum of ApEn to capture the complexity adequately^46^, including *N* ≃ 8760 data points for each analyzed year. The result of both algorithms is a real number with lower values indicating more predictability. Formally, the steps for calculating ApEn are summarized as:

Step 1. First, it is required to fix the length of the compared patterns (embedding dimension) *m*, and the effective noise filter, *r*.

Step 2. Given a data series {*u*(*i*)} of length *N*, define the sequences of length *m* $x(i)=[u(i),\ldots ,u(i+m-1)]$ and $x(j)=[u(j),\ldots ,u(j+m-1)]$, with *i* being the vector acting as template and with *j* being the rest of the subsequences of the data series. Then, determine the distance $d[x(i),x(j)]$ as the maximum distance of the scalar components for each vector acting as template once.

Step 3. Determine the correlation integrals, i.e., the regularity or frequency, within a tolerance *r*, of patterns similar to a given pattern of length *m*. Using the sequences $x(i)=[x(1),x(2),\ldots ,x(N-m+1)]$ for *i* ≤ *N* − *m* + 1, calculate ${C}_{i}^{m}(r)=$ (number of *j* ≤ *N*-*m* + 1 for which *d*[*x*(*i*), *x*(*j*)] ≤ *r*)/(*N* − *m* + 1).

Step 4. Define the functions ${\phi }^{m}(r)=(N-m+1){\sum }_{i=1}^{N-m+1}\,\mathrm{ln}\,{C}_{i}^{m}(r)$

Step 5. Determine the likelihood that runs of patterns that are close for *m* observations remain close on the next incremental comparisons, defining the negative value of ApEn as:

- ${\rm{ApEn}}={\phi }^{m+1}(r)-{\phi }^{m}(r)$ = average over *i* of ln [conditional probability that $|u(i+m)-u(j+m)|\le r$, given the fact that the previous values fulfill the condition $|u(i+k)-u(j+k)|\le r$ for $k=0,1,\ldots ,m-1$].

### Sample Entropy

This algorithm is a variation of ApEn which makes direct use of the correlation integral, designed to eliminate the bias induced by self counting^20^; note the $i\ne j$ restriction in the sums. Both algorithms are based on the calculation of conditional probabilities, and the first two steps are similar to ApEn.

Step 3. Calculate, for each template vector, $${B}_{i}^{m}(r)=\frac{1}{N-m-1}{\sum }_{j=1,j\ne i}^{N-m}$$ $$[{\rm{number}}\,{\rm{of}}\,{\rm{times}}\,{\rm{that}}$$$$d[{x}_{m}(j)-{x}_{m}(i)] < r]$$. Then sum over all template vectors $${B}^{m}(r)=\frac{1}{N-m}{\sum }_{i=1}^{N-m}{B}_{i}^{m}(r)$$.

Step 4. Similarly, for each template vector, determine $${A}_{i}^{m}(r)=\frac{1}{N-m-1}{\sum }_{j=1,j\ne i}^{N-m}$$ $$[{\rm{number}}\,{\rm{of}}\,{\rm{times}}\,{\rm{that}}$$$$d[|{x}_{m+1}(j)-{x}_{m+1}(i)|] < r]$$ and the sum over all template vectors $${A}^{m}(r)=\frac{1}{N-m}{\sum }_{i=1}^{N-m}{A}_{i}^{m}(r)$$

Step 5. Sample Entropy is determined as SampEn(*m*,*r*,*N*) = −log[*A*^*m*^(*r*)/*B*^*m*^(*r*)].

### Transfer entropy

In Information Theory, entropy is a magnitude which quantifies the information content in a data series^47^. Considering a set of possible events whose probabilities of occurrence are *p*_*i*_, $i=\{1,\ldots ,n\}$, then the entropy $H({p}_{1},{p}_{2},\ldots ,{p}_{n})$ is a measure of the uncertainty of the outcome of an event given such distribution, determined as $H=-\,{\sum }_{i=1}^{N}{p}_{i}{\log }_{2}{p}_{i}$.

Based on that measure, the Mutual Information of two processes *I* and *J* whose joint probability is *p*_*IJ*_ (*i*, *j*) is defined as the amount of information obtained about one variable through observing the other, ${M}_{IJ}=\sum p(i,j)\log \,\frac{p(i,j)}{p(i)p(j)}$. *M*_*IJ*_ is symmetric and it does not contain any directional sense, being a correlation measure. However, this measure is useful for determining the number of previous values which affect the current value of a data series, by comparing each series with itself but lagged a different number of periods. We choose the number of lags which makes the Mutual Information between a series and its lagged versions decrease the most, to assure that it is independent from itself with a delay of *k*. For both SW and LW radiation we obtain that the previous two values have the most influence on the next value, since values of *k* > 2 barely decrease the Mutual Information (see Supplementary Material).

To take into account the directionality of the information flux, the Transfer Entropy^33^ from a variable *Y* to a variable *X* is defined as:

$$T{E}_{Y\to X}(k,l)=\sum p({i}_{t+1},{i}_{t}^{(k)},{j}_{t}^{(l)}){\log }_{2}\frac{p({i}_{t+1}|{i}_{t}^{(k)},{j}_{t}^{(l)})}{p({i}_{t+1}|{i}_{t}^{(k)})},$$

where *i*_*t*_ is the element *t* of the variable *X* and *j*_*t*_ is the element *t* of the variable *Y*; *p*(*A, B*) denotes the joint probability of two events *A* and *B*; and $p({i}_{t+1},{i}_{t}^{(k)},{j}_{t}^{(l)})$ denotes the joint probability distribution of state *i*_*t*+1_ with its *k* + 1 predecessors and the *l* predecessors of sate *j*_*t*_, formally $p({i}_{t+1},{i}_{t}^{(k)},{j}_{t}^{(l)})=p({i}_{t+1},{i}_{t},\ldots ,{i}_{t-k+1},{j}_{t},\ldots ,{j}_{t-l+1})$. From the calculations of the Mutual Information, we obtained a lag of two hours for shortwave (*k* = 2) and longwave (*l* = 2) radiation. As we are working with hourly data, it means that the two previous hours are enough to predict the next hour, and adding more information about previous values of the time series does not increase much our information for the prediction.

In order to calculate Transfer Entropy, a stationary set of data and a discrete partition are required. We follow a symbolic encoding approach for partitioning the data automatically depending on the percentiles *q*_1_ = 0.05 and *q*_2_ = 0.95 for each of the analyzed years. As Transfer Entropy is asymmetric, $T{E}_{SW\to LW}(k,l)\ne T{E}_{LW\to SW}(k,l)$, we can analyze the changes in information fluxes between SW and LW radiation in the last four decades. To eliminate the small sample effects, we determine the Effective Transfer Entropy to eliminate those relations between the variables which could be due to coincidences instead of real non-linear interactions, $ET{E}_{J\to I}={T}_{J\to I}(k,l)-{T}_{{J}_{shuffled}(k,l)\to I}$. We use a procedure introduced by Dimpfl and Peter^48^ which outperforms random shuffling in small samples by bootstraping the underlying Markov process, destroying the dependence between the variables but retaining the dynamics of the series. For the calculations of Transfer Entropy we rely on their R package^49^, proving that all the calculations from SW to LW are statistically significant with p-values < 0.001, while some years from LW are less significant or even not statistically significant.

Our interpretation of this fact is that shortwave radiation is the main driver in the dynamics and plays a major role in the energy balance by affecting the longwave radiation field, while the information flux from longwave to shortwave radiation is marginal. As discussed above, shortwave radiation is directly reflected by clouds or surface for example, while longwave radiation is reemitted after absorption of the incoming solar radiation. Those different timescales are of importance in understanding the dynamics; changes in cloud fraction for example would be rapidly manifested in the shortwave field and would influence the longwave radiation field since the available energy for being absorbed would be different. Some geoengineering suggestions to cool down the planet by placing reflective particles in the atmosphere rely on this mechanism. On the contrary, changes in the longwave radiation field are much slower and their influence in the shortwave flux is indirect by affecting the whole atmospheric and oceanic systems and modifying the patterns in the water cycle for example. The results will be discussed in the next section, and the Supplementary Material includes the individualized results of the statistical significance of each year.

## Results

### Complexity analysis

Figures 1 and 2 show the shortwave and longwave radiation record from the MERRA-2 reanalysis along with the usual measures of moment statistics, namely mean, variance, skewness, and kurtosis. We can see that, in both cases, the mean and kurtosis have a positive trend, while the variance and skewness trend have a different sign for both kinds of radiation, being the *x*– axis the number of years since 1980. To study the evolution, variability measures can detect deviations from the average value but do not worry about the regularity of the data. The best analysis of a data series would be the combined use of regularity statistics and moment statistics^50^.

In this sense, ApEn has been shown to distinguish normal from abnormal data in instances where moment statistic approaches failed to show significant differences^51^. We analyze the complexity of the shortwave and longwave radiation data individually with ApEn and SampEn to study the changes in their complexity through time. Both algorithms are based on counting the different number of patterns and their repetitions in the dataset. The main difference is that SampEn does not allow self counting and uses the whole series at the same time, requiring only that a template vector find a match to be defined, while ApEn allows self counting and requires each template vector to find a match to be defined. In both cases, higher values indicate higher randomness and low predictability. In order to use the algorithms, it is mandatory to make the series stationary^24^. A common way to analyse the evolution of the changes without assuming a normal distribution of the variations is to first transform the series by taking the logarithm to stabilize the variance and then subtract the previous value of the logarithm to eliminate the trend, log (*x*_*t*_/*x*_*t*–1_)^48,52^, inducing stationarity.

**Figure 3:**
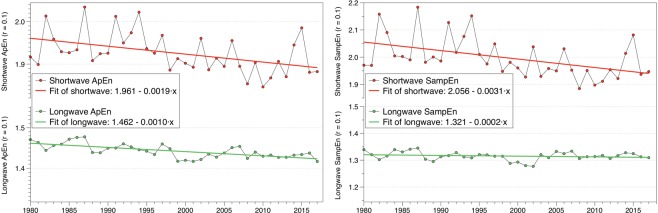
Complexity analysis of shortwave and longwave radiation using Approximate Entropy (left) and Sample Entropy (right) using an embedding dimension *m* = 2 and a tolerance *r* = 0.1σ.

Both ApEn and SampEn are relative algorithms, meaning that comparisons must be done between similar sequences. We analyze the complexity of SW and LW hourly radiation data separately on a yearly basis to study the behavior in the last 37 years. Figure 3 shows the results for Approximate Entropy (left) and Sample Entropy (right), along with the least square fits for the last four decades. Both algorithms confirm a decreasing trend in complexity for SW radiation and a much smaller trend for LW radiation. The statistical parameters of the fits and a SW radiation analysis with different embedding dimensions to confirm the robustness of the trend are included in the Supplementary Material.

The figure shows that SW and LW radiation have changed in a different manner in the last four decades, being the changes in SW more noticeable. Hence, as both kinds of radiation are linked through the energy balance of the Earth’ system, we would expect changes in their coupled dynamics.

### Causality

To analyze the causality and directionality of those changes, we study the information flow between them using the Effective Transfer Entropy (ETE). ETE is determined to provide more statistical significance by discerning between real information flows and the statistical artifacts which could occur due to small sample effects. Instead of merely shuffling the values to estimate the bias, the bootstrapping procedure of ETE provides a more accurate calculation of the distribution of the transfer entropy estimates under the assumption of no information flow while retaining the dynamics of the univariate time series^48^. This fact allows us to calculate not only the value of the information flux but also the p-values of the significance. For using ETE, we determine the proper embedding parameters using a Mutual Information analysis^45^, finding a lag of *k* = 2 for SW and *l* = 2 for LW.

**Figure 4:**
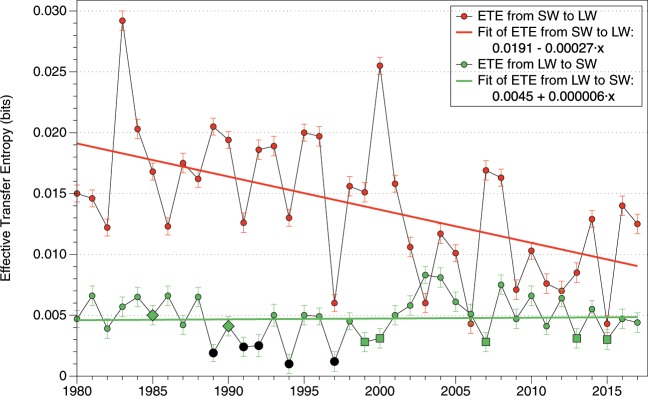
Effective Transfer Entropy fluxes between shortwave and longwave radiation during the last four decades in a yearly basis. The statistical significance is determined by the p-values: <0.001=◦, <0.01=◊, <0.1= □, and not-significant =●.

Figure 4 shows the ETE for the last 37 years on a yearly basis and the least square fit for each kind of radiation. Each point quantifies the yearly information flux from SW to LW (red) and from LW to SW (green), indicating the statistical significance of the transference as specified in the caption of the figure. The Mann-Kendall trend test confirmed the SW monotonic variations, but it must be noticed that the actual trend may or may not be linear. The results show that the importance of SW in LW radiation is higher, always significant and has decreased during this period, while the information flow from LW to SW has been almost constant and not significant in many occasions.

Both the individual series complexity analysis with ApEn and SampEn and the coupled dynamic causality analysis with Transfer Entropy support the idea that SW radiation has changed the most. SW radiation is mainly determined by clouds and surface albedo, suggesting that the changes in those parameters are of great importance for recent climate change. The timescales of variation for SW and LW radiation are very different. For SW, changes in clouds for example would change the radiation field quickly. However, for LW radiation the incoming solar radiation needs to be absorbed first and reemitted afterwards, being a slower process. Shortwave and longwave radiation are related through the energy balance, and the incoming solar radiation it is either reflected (SW) or absorbed (LW). The existence of reflective surfaces such as clouds or the ground determines directly the amount of SW radiation and, thus, the available energy for being absorbed and reemitted as LW. On the other hand, the increase of the temperature of the planet for example has an effect on the cloud coverage as well, explaining the transference of information from longwave to shortwave.

**Figure 5:**
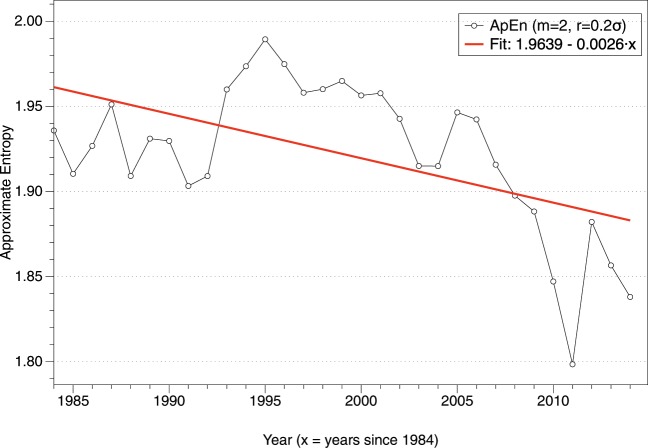
ApEn(m = 2, *r* = 0.2σ, N ≃ 2920) for every year of ISCCP data from 1984 to 2014.

A plausible hypothesis to explain the changes in SW radiation is that cloud amount and properties have changed in the last decades. Previous research has investigated those changes, focusing on specific types of clouds^16^ or global scale trends^53^, quantifying the decreasing rate of change of cloud fraction using different satellite-derived cloud climate data records^54^. Using source product HGG of the International Satellite Cloud Climatology Project (ISCCP)^43^, we analyze the changes in complexity in cloud amount globally every 3 hours after making the series stationary to avoid problems with trends and cycles. This analysis is different from variability of cloud amount and focuses on complexity using Approximate Entropy. Figure 5 shows the evolution of cloud amount complexity using ISCCP data set from 1984 to 2014 in a yearly basis, showing a decreasing trend which indicates changes in cloud amount behavior towards more predictability.

## Discussion and Conclusions

The thermodynamic state of the Earth is maintained by a balance between incoming solar radiation and outgoing shortwave and longwave radiation from Earth; changes in the outgoing radiation field generate an energy imbalance which is the driver of current climate change.

In this paper, we have analyzed the complexity and causality of the shortwave and longwave radiation fields using global hourly data from MERRA-2. Using Approximate Entropy and Sample Entropy, we showed that the predictability of SW radiation is changing faster than for LW radiation. In our work, we have prescinded of any trends or cycles in the data by forcing stationarity after a log-ratio transformation to capture the differences in complexity. We interpret these differences in the view of different timescales. Changes in clouds for example would have an immediate representation in the shortwave field but the global warming affecting the longwave field takes much longer in comparison. Clouds and surface albedo have changed in the last four decades, as reported in previous research, and those differences should be reflected in changes in the dynamic system between SE and LW radiation. Consequently, we evaluated causality to study the evolution of those changes.

Due to its complex nature, the relation between SW and LW cannot be assumed to be linear, and classic techniques such as momentum statistics or Granger causality fail to capture the whole evolution of its complexity. According to our analysis using Transfer Entropy, SW radiation has more influence on the system, being the fluxes of information from SW to LW larger and decreasing in the last decades. The transfer of information from LW to SW is minimal, being some years not statistically significant and peaking around 2002, a fact which we have not been able to explain in our analysis. This decoupling between shortwave and longwave radiation indicates that the dynamics of the climatic system in the last 40 years has changed, as observed in the energy imbalance of the last decades, suggesting that changes in SW played a mayor role.

Our research supports the idea that clouds and albedo, which ultimately determine the SW radiation, are variables of the utmost importance for current climate change, in agreement with previous research about the changes in stratocumulus or energy imbalance in the last four decades for example. An increase in cloud coverage of 0.1 would, on average, lead to a 7% increase in spectrally integrated global average reflectance of shortwave radiation^55^. The Decadal Survey for Earth Science and Applications from Space (2018) lists as one of the key science questions “how changing cloud cover and precipitation will affect climate, weather and Earth’s energy balance in the future”. We investigated the hypothesis that changes in clouds are responsible for the changes in SW radiation by calculating the evolution of complexity of cloud amount using ISCCP data, showing an increase in predictability and lower ApEn values, similarly to SW radiation. Even though the hypothesis of variations in cloud amount could explain the changes in SW radiation and the changes observed in the Transfer Entropy analysis, it is not possible to guarantee that clouds are the only factor since climate is a highly interconnected system, as reflected in the complexity of General Circulation Models.

## Supplementary information

Supplementary Information.
