# Supplementary material for: Analyzing changes in the complexity of climate in the last four decades using MERRA-2 radiation data
Source: Sci Rep. 2020 Jan 22;10:922. doi: 10.1038/s41598-020-57917-8 (PMC6976651; doi:10.1038/s41598-020-57917-8)
Supplement: Supplementary file 1 — Supplementary Information. [file 41598_2020_57917_MOESM1_ESM.pdf]

Analyzing changes in the complexity of climate in the last four decades using MERRA-2 radiation data

Supplementary Material

Alfonso Delgado-Bonal[1,2,*], Alexander Marshak[2], Yuekui Yang[2] and Daniel Holdaway[3]

[1]NASA Goddard Space Flight Center, Greenbelt, Maryland. [2]Universities Space Research Association, Columbia, Maryland. [3]NASA Goddard Space Flight Center, Global Modeling and Assimilation Office, Greenbelt, MD

* alfonso.delgadobonal@nasa.gov

Index

1 . Momentum Statistics 2
2 . ApEn and SampEn Results 3
3 . Mutual Information Results 4
4 . Transfer Entropy Results 5
4.1 Effective Transfer Entropy four decades fit 5
4.2 Yearly Transfer Entropy calculations 5-25

1- Momentum Statistics

SW	Mean	Variance	Skewness	Kurtosis
FIT	103.332 + 0.0998342*x	29.097 + 0.38949*x	0.0935 + 0.004044*x	-0.6754 + 0.0004639*x
Intercept	103.332	29.097	0.0935	-0.6754
σ_Intercept	0.304571	1.0622	0.03711	0.04788
X Intercept	-1035.04	-74.705	-23.12	1456
Slope	0.0998342	0.38949	0.004044	0.0004639
σ_Slope	0.013614	0.047479	0.001659	0.00214
Correlation	0.778298	0.81109	0.381	0.03662
R^2	0.605748	0.65787	0.1452	0.001341
σ	0.883207	3.0802	0.1076	0.1388
s for residual	0.895063	3.1215	0.1091	0.1407
Sample Size	38	38	38	38

Table A.1 – Momentum statistics table for short-wave radiation data from 1980-2017.

LW	Mean	Variance	Skewness	Kurtosis
FIT	237.46 + 0.021439*x	6.9047 - 0.017996*x	0.29496 - 0.0011717*x	-0.70326 + 0.0030776*x
Intercept	237.46	6.9047	0.29496	-0.70326
σ_Intercept	0.17769	0.42193	0.049873	0.076969
X Intercept	-11076	383.69	251.74	228.51
Slope	0.021439	-0.017996	-0.0011717	0.0030776
σ_Slope	0.0079425	0.01886	0.0022293	0.0034404
Correlation	0.4151	-0.15923	-0.08849	0.1495
R^2	0.17231	0.025354	0.0078305	0.022351
σ	0.51527	1.2235	0.14463	0.2232
s for residual	0.52218	1.24	0.14657	0.22619
Sample Size	38	38	38	38

Table A.2 – Momentum statistics table for long-wave radiation data from 1980-2017.

2- ApEn and SampEn Results

	ApEn SW	ApEn LW	SampEn SW	SampEn LW
FIT	1.9609 - 0.0018711*x	1.462 - 0.0010405*x	1.9796 - 5.929e-11*x	1.4724 - 3.2972e-11*x
Intercept	1.9609	1.462	1.9796	1.4724
σ_Intercept	0.013524	0.0040316	0.019202	0.0057245
X Intercept	1048	1405	3.3389e10	4.4656e10
Slope	-0.0018711	-0.0010405	-5.929e-11	-3.2972e-11
σ_Slope	0.00062884	0.00018746	1.9927e-11	5.9404e-12
Correlation	-0.44932	-0.68421	-0.44931	-0.68421
R^2	0.20188	0.46815	0.20188	0.46814
σ	0.040796	0.012162	0.040796	0.012162
s for residual	0.041343	0.012325	0.041343	0.012325
Sample Size	38	38	38	38

Table A.3 – ApEn and SampEn fit table for long-wave radiation data from 1980-2017 for m = 2.

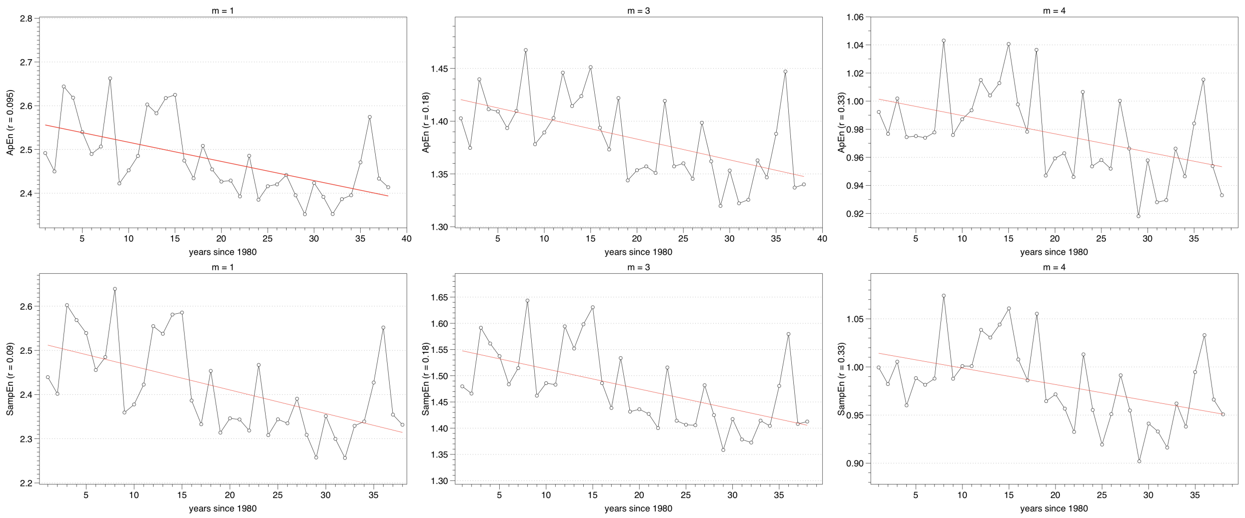

Figure A.1 – ApEn and SampEn for SW radiation from 1980-2017 using different values of the embedding dimension m = 1,3, and 4 to confirm the trend

3- Mutual Information Results

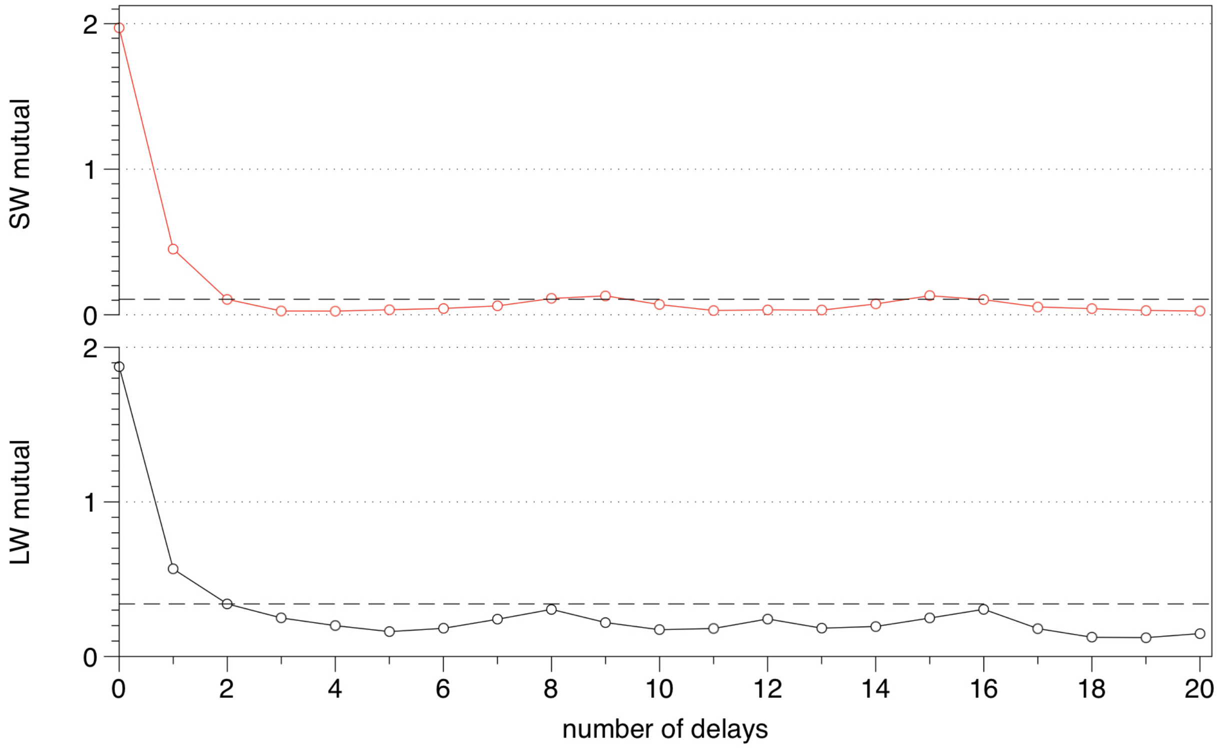

Figure A.2 – Mutual Information analysis for short- and long-wave radiation.

Mutual information is calculated as:

$$M_{\{I,J\}} = \sum p(i,j) \log \frac{p(i,j)}{p(i)p(j)}$$

We compare the series of short-wave (Figure A.2 - top) and long-wave (Figure A.2 - bottom) radiation with lagged versions of themselves. If the considered number of delays is insufficient, then the results of the Transfer Entropy analysis may be based on previous results of the series, instead of information flux from other series. If the number of delays is too large, we are introducing useless information which may be considered as transference of information which is not due to the dynamics and decrease the statistical significance of the Transfer Entropy results. We select a number of lags of two for both series (the two previous hours have influence in the prediction of the next value). The gain of information by adding more delays is minimal an basically independent of the number of delays.

4- Transfer Entropy Results

4.1 – Effective Transfer Entropy four decades fit

	SW to LW	LW to SW
FIT	0.019111 - 0.00027216*x	0.004593 + 6.795e-6*x
Intercept	0.019111	0.004593
σ_Intercept	0.0015573	0.0005856
X Intercept	70.22	-675.9
Slope	-0.00027216	6.795e-6
σ_Slope	7.2414e-5	2.723e-5
Correlation	-0.53623	0.04214
R^2	0.28754	0.001776
σ	0.0046979	0.001767
s for residual	0.0047609	0.00179
Sample Size	38	38

Table A.4 – Fit of ETE between short- and long-wave radiation.

4.2 –Yearly Transfer Entropy calculations

"1980-01-01 GMT" to "1980-12-31 GMT"

```
Shannon's entropy on 8 cores with 100 shuffles.
x and y have length 8783 (0 NAs removed)
[calculate] X->Y transfer entropy
[calculate] Y->X transfer entropy
[bootstrap] 300 times
Done - Total time 25.75 seconds
Shannon Transfer Entropy Results:
----------------------------------------------------------
Direction       TE   Eff. TE  Std.Err.   p-value    sig
----------------------------------------------------------
X->Y    0.0192    0.0150    0.0007    0.0000    ***
Y->X    0.0085    0.0047    0.0007    0.0000    ***
----------------------------------------------------------
Bootstrapped TE Quantiles (300 replications):
----------------------------------------------------------
Direction     0%     25%     50%     75%    100%
----------------------------------------------------------
X->Y  0.0031  0.0045  0.0048  0.0053  0.0070
Y->X  0.0024  0.0040  0.0045  0.0050  0.0072
----------------------------------------------------------
Number of Observations: 8783
----------------------------------------------------------
p-values: < 0.001 '***', < 0.01 '**', < 0.05 '*', < 0.1 '.'
```

"1981-01-01 GMT" to "1981-12-31 GMT"

```
Shannon's entropy on 8 cores with 100 shuffles.
x and y have length 8760 (0 NAs removed)
[calculate] X->Y transfer entropy
[calculate] Y->X transfer entropy
[bootstrap] 300 times
Done - Total time 25.68 seconds
Shannon Transfer Entropy Results:
---------------------------------------------------------
Direction       TE   Eff. TE  Std.Err.   p-value    sig
---------------------------------------------------------
X->Y    0.0189    0.0146    0.0007    0.0000    ***
Y->X    0.0103    0.0066    0.0008    0.0000    ***
---------------------------------------------------------
Bootstrapped TE Quantiles (300 replications):
---------------------------------------------------------
Direction     0%     25%     50%     75%    100%
---------------------------------------------------------
X->Y  0.0034  0.0045  0.0049  0.0054  0.0083
Y->X  0.0032  0.0042  0.0046  0.0050  0.0066
---------------------------------------------------------
Number of Observations: 8760
---------------------------------------------------------
p-values: < 0.001 '***', < 0.01 '**', < 0.05 '*', < 0.1 '.'
```

"1982-01-01 GMT" to "1982-12-31 GMT"

```
Shannon's entropy on 8 cores with 100 shuffles.
x and y have length 8760 (0 NAs removed)
[calculate] X->Y transfer entropy
[calculate] Y->X transfer entropy
[bootstrap] 300 times
Done - Total time 25.56 seconds
Shannon Transfer Entropy Results:
---------------------------------------------------------
Direction       TE   Eff. TE  Std.Err.   p-value    sig
---------------------------------------------------------
X->Y    0.0165    0.0122    0.0007    0.0000    ***
Y->X    0.0081    0.0039    0.0008    0.0000    ***
---------------------------------------------------------
Bootstrapped TE Quantiles (300 replications):
---------------------------------------------------------
Direction     0%     25%     50%     75%    100%
---------------------------------------------------------
X->Y  0.0031  0.0045  0.0050  0.0055  0.0076
Y->X  0.0030  0.0043  0.0046  0.0052  0.0070
---------------------------------------------------------
Number of Observations: 8760
```

```
---------------------------------------------------------
p-values: < 0.001 '***', < 0.01 '**', < 0.05 '*', < 0.1 '.'
```

"1983-01-01 GMT" to "1983-12-31 GMT"

```
Shannon's entropy on 8 cores with 100 shuffles.
x and y have length 8760 (0 NAs removed)
[calculate] X->Y transfer entropy
[calculate] Y->X transfer entropy
[bootstrap] 300 times
Done - Total time 24.97 seconds
Shannon Transfer Entropy Results:
---------------------------------------------------------
Direction       TE   Eff. TE  Std.Err.   p-value    sig
---------------------------------------------------------
X->Y    0.0335    0.0292    0.0008    0.0000    ***
Y->X    0.0094    0.0057    0.0008    0.0000    ***
---------------------------------------------------------
Bootstrapped TE Quantiles (300 replications):
---------------------------------------------------------
Direction     0%     25%     50%     75%    100%
---------------------------------------------------------
X->Y  0.0031  0.0047  0.0053  0.0059  0.0079
Y->X  0.0029  0.0042  0.0048  0.0054  0.0077
---------------------------------------------------------
Number of Observations: 8760
---------------------------------------------------------
p-values: < 0.001 '***', < 0.01 '**', < 0.05 '*', < 0.1 '.'
```

"1984-01-01 GMT" to "1984-12-31 GMT"

```
Shannon's entropy on 8 cores with 100 shuffles.
x and y have length 8784 (0 NAs removed)
[calculate] X->Y transfer entropy
[calculate] Y->X transfer entropy
[bootstrap] 300 times
Done - Total time 24.94 seconds
Shannon Transfer Entropy Results:
---------------------------------------------------------
Direction       TE   Eff. TE  Std.Err.   p-value    sig
---------------------------------------------------------
X->Y    0.0245    0.0203    0.0008    0.0000    ***
Y->X    0.0104    0.0065    0.0008    0.0000    ***
---------------------------------------------------------
Bootstrapped TE Quantiles (300 replications):
---------------------------------------------------------
Direction     0%     25%     50%     75%    100%
---------------------------------------------------------
X->Y  0.0032  0.0043  0.0048  0.0053  0.0088
Y->X  0.0031  0.0041  0.0046  0.0052  0.0068
```

```
----------------------------------------------------------
Number of Observations: 8784
----------------------------------------------------------
p-values: < 0.001 '***', < 0.01 '**', < 0.05 '*', < 0.1 '.'
```

"1985-01-01 GMT" to "1985-12-31 GMT"

```
Shannon's entropy on 8 cores with 100 shuffles.
x and y have length 8760 (0 NAs removed)
[calculate] X->Y transfer entropy
[calculate] Y->X transfer entropy
[bootstrap] 300 times
Done - Total time 24.9 seconds
Shannon Transfer Entropy Results:
----------------------------------------------------------
Direction      TE   Eff. TE  Std.Err.  p-value   sig
----------------------------------------------------------
X->Y   0.0212   0.0168   0.0007   0.0000   ***
Y->X   0.0090   0.0050   0.0008   0.0033    **
----------------------------------------------------------
Bootstrapped TE Quantiles (300 replications):
----------------------------------------------------------
Direction    0%     25%     50%     75%    100%
----------------------------------------------------------
X->Y  0.0031  0.0045  0.0050  0.0055  0.0096
Y->X  0.0027  0.0040  0.0045  0.0049  0.0065
----------------------------------------------------------
Number of Observations: 8760
----------------------------------------------------------
p-values: < 0.001 '***', < 0.01 '**', < 0.05 '*', < 0.1 '.'
```

"1986-01-01 GMT" to "1986-12-31 GMT"

```
Shannon's entropy on 8 cores with 100 shuffles.
x and y have length 8760 (0 NAs removed)
[calculate] X->Y transfer entropy
[calculate] Y->X transfer entropy
[bootstrap] 300 times
Done - Total time 24.9 seconds
Shannon Transfer Entropy Results:
----------------------------------------------------------
Direction      TE   Eff. TE  Std.Err.  p-value   sig
----------------------------------------------------------
X->Y   0.0165   0.0123   0.0007   0.0000   ***
Y->X   0.0103   0.0066   0.0008   0.0000   ***
----------------------------------------------------------
Bootstrapped TE Quantiles (300 replications):
----------------------------------------------------------
Direction    0%     25%     50%     75%    100%
----------------------------------------------------------
```

```
X->Y  0.0031  0.0045  0.0051  0.0056  0.0079
Y->X  0.0029  0.0042  0.0047  0.0052  0.0068
---------------------------------------------------------
Number of Observations: 8760
---------------------------------------------------------
p-values: < 0.001 '***', < 0.01 '**', < 0.05 '*', < 0.1 '.'
```

"1987-01-01 GMT" to "1987-12-31 GMT"

```
Shannon's entropy on 8 cores with 100 shuffles.
x and y have length 8760 (0 NAs removed)
[calculate] X->Y transfer entropy
[calculate] Y->X transfer entropy
[bootstrap] 300 times
Done - Total time 24.98 seconds
Shannon Transfer Entropy Results:
---------------------------------------------------------
Direction       TE   Eff. TE  Std.Err.   p-value    sig
---------------------------------------------------------
X->Y    0.0218    0.0175    0.0008    0.0000    ***
Y->X    0.0083    0.0042    0.0008    0.0000    ***
---------------------------------------------------------
Bootstrapped TE Quantiles (300 replications):
---------------------------------------------------------
Direction     0%     25%     50%     75%    100%
---------------------------------------------------------
X->Y  0.0033  0.0044  0.0050  0.0054  0.0082
Y->X  0.0030  0.0041  0.0046  0.0053  0.0070
---------------------------------------------------------
Number of Observations: 8760
---------------------------------------------------------
p-values: < 0.001 '***', < 0.01 '**', < 0.05 '*', < 0.1 '.'
```

"1988-01-01 GMT" to "1988-12-31 GMT"

```
Shannon's entropy on 8 cores with 100 shuffles.
x and y have length 8784 (0 NAs removed)
[calculate] X->Y transfer entropy
[calculate] Y->X transfer entropy
[bootstrap] 300 times
Done - Total time 25.16 seconds
Shannon Transfer Entropy Results:
---------------------------------------------------------
Direction       TE   Eff. TE  Std.Err.   p-value    sig
---------------------------------------------------------
X->Y    0.0205    0.0162    0.0007    0.0000    ***
Y->X    0.0105    0.0065    0.0008    0.0000    ***
---------------------------------------------------------
Bootstrapped TE Quantiles (300 replications):
---------------------------------------------------------
```

```
Direction     0%     25%     50%     75%    100%
-------------------------------------------------------
X->Y  0.0032  0.0045  0.0050  0.0056  0.0072
Y->X  0.0029  0.0041  0.0045  0.0049  0.0077
-------------------------------------------------------
Number of Observations: 8784
-------------------------------------------------------
p-values: < 0.001 '***', < 0.01 '**', < 0.05 '*', < 0.1 '.'
```

"1989-01-01 GMT" to "1989-12-31 GMT"

```
Shannon's entropy on 8 cores with 100 shuffles.
x and y have length 8760 (0 NAs removed)
[calculate] X->Y transfer entropy
[calculate] Y->X transfer entropy
[bootstrap] 300 times
Done - Total time 26.65 seconds
Shannon Transfer Entropy Results:
-------------------------------------------------------
Direction       TE   Eff. TE  Std.Err.  p-value    sig
-------------------------------------------------------
X->Y    0.0250    0.0205    0.0007    0.0000    ***
Y->X    0.0058    0.0019    0.0007    0.1633
-------------------------------------------------------
Bootstrapped TE Quantiles (300 replications):
-------------------------------------------------------
Direction     0%     25%     50%     75%    100%
-------------------------------------------------------
X->Y  0.0034  0.0045  0.0051  0.0055  0.0076
Y->X  0.0034  0.0043  0.0048  0.0052  0.0069
-------------------------------------------------------
Number of Observations: 8760
-------------------------------------------------------
p-values: < 0.001 '***', < 0.01 '**', < 0.05 '*', < 0.1 '.'
```

"1990-01-01 GMT" to "1990-12-31 GMT"

```
Shannon's entropy on 8 cores with 100 shuffles.
x and y have length 8760 (0 NAs removed)
[calculate] X->Y transfer entropy
[calculate] Y->X transfer entropy
[bootstrap] 300 times
Done - Total time 25.94 seconds
Shannon Transfer Entropy Results:
-------------------------------------------------------
Direction       TE   Eff. TE  Std.Err.  p-value    sig
-------------------------------------------------------
X->Y    0.0239    0.0194    0.0007    0.0000    ***
Y->X    0.0078    0.0041    0.0008    0.0033     **
-------------------------------------------------------
```

```
Bootstrapped TE Quantiles (300 replications):
--------------------------------------------------------
Direction     0%     25%     50%     75%    100%
--------------------------------------------------------
X->Y  0.0032  0.0044  0.0048  0.0053  0.0081
Y->X  0.0031  0.0042  0.0048  0.0054  0.0069
--------------------------------------------------------
Number of Observations: 8760
--------------------------------------------------------
p-values: < 0.001 '***', < 0.01 '**', < 0.05 '*', < 0.1 '.'
```

```
"1991-01-01  GMT" to "1991-12-31 GMT"
Shannon's entropy on 8 cores with 100 shuffles.
x and y have length 8760 (0 NAs removed)
[calculate] X->Y transfer entropy
[calculate] Y->X transfer entropy
[bootstrap] 300 times
Done - Total time 26.32 seconds
Shannon Transfer Entropy Results:
--------------------------------------------------------
Direction       TE   Eff. TE  Std.Err.   p-value    sig
--------------------------------------------------------
X->Y    0.0169    0.0126    0.0008    0.0000    ***
Y->X    0.0062    0.0024    0.0008    0.0900     .
--------------------------------------------------------
Bootstrapped TE Quantiles (300 replications):
--------------------------------------------------------
Direction     0%     25%     50%     75%    100%
--------------------------------------------------------
X->Y  0.0032  0.0045  0.0050  0.0056  0.0077
Y->X  0.0030  0.0044  0.0049  0.0054  0.0080
--------------------------------------------------------
Number of Observations: 8760
--------------------------------------------------------
p-values: < 0.001 '***', < 0.01 '**', < 0.05 '*', < 0.1 '.'
```

```
"1992-01-01  GMT" to "1992-12-31 GMT"
Shannon's entropy on 8 cores with 100 shuffles.
x and y have length 8784 (0 NAs removed)
[calculate] X->Y transfer entropy
[calculate] Y->X transfer entropy
[bootstrap] 300 times
Done - Total time 25.43 seconds
Shannon Transfer Entropy Results:
--------------------------------------------------------
Direction       TE   Eff. TE  Std.Err.   p-value    sig
--------------------------------------------------------
X->Y    0.0230    0.0186    0.0008    0.0000    ***
```

```
Y->X    0.0065    0.0025    0.0009    0.1100
---------------------------------------------------------
Bootstrapped TE Quantiles (300 replications):
---------------------------------------------------------
Direction     0%     25%     50%     75%    100%
---------------------------------------------------------
X->Y  0.0035  0.0048  0.0052  0.0059  0.0078
Y->X  0.0027  0.0043  0.0047  0.0053  0.0078
---------------------------------------------------------
Number of Observations: 8784
---------------------------------------------------------
p-values: < 0.001 '***', < 0.01 '**', < 0.05 '*', < 0.1 '.'
```

"1993-01-01 GMT" to "1993-12-31 GMT"

```
Shannon's entropy on 8 cores with 100 shuffles.
x and y have length 8760 (0 NAs removed)
[calculate] X->Y transfer entropy
[calculate] Y->X transfer entropy
[bootstrap] 300 times
Done - Total time 26.73 seconds
Shannon Transfer Entropy Results:
---------------------------------------------------------
Direction       TE   Eff. TE  Std.Err.   p-value    sig
---------------------------------------------------------
X->Y    0.0232    0.0189    0.0008    0.0000    ***
Y->X    0.0097    0.0050    0.0009    0.0000    ***
---------------------------------------------------------
Bootstrapped TE Quantiles (300 replications):
---------------------------------------------------------
Direction     0%     25%     50%     75%    100%
---------------------------------------------------------
X->Y  0.0034  0.0052  0.0058  0.0064  0.0085
Y->X  0.0031  0.0043  0.0048  0.0054  0.0070
---------------------------------------------------------
Number of Observations: 8760
---------------------------------------------------------
p-values: < 0.001 '***', < 0.01 '**', < 0.05 '*', < 0.1 '.'
```

"1994-01-01 GMT" to "1994-12-31 GMT"

```
Shannon's entropy on 8 cores with 100 shuffles.
x and y have length 8760 (0 NAs removed)
[calculate] X->Y transfer entropy
[calculate] Y->X transfer entropy
[bootstrap] 300 times
Done - Total time 25.29 seconds
Shannon Transfer Entropy Results:
---------------------------------------------------------
Direction       TE   Eff. TE  Std.Err.   p-value    sig
```

```
---------------------------------------------------------
X->Y   0.0173   0.0130   0.0007   0.0000   ***
Y->X   0.0051   0.0010   0.0008   0.4700
---------------------------------------------------------
Bootstrapped TE Quantiles (300 replications):
---------------------------------------------------------
Direction     0%     25%     50%     75%    100%
---------------------------------------------------------
X->Y  0.0031  0.0046  0.0051  0.0057  0.0075
Y->X  0.0031  0.0043  0.0048  0.0053  0.0069
---------------------------------------------------------
Number of Observations: 8760
---------------------------------------------------------
p-values: < 0.001 '***', < 0.01 '**', < 0.05 '*', < 0.1 '.'
```

"1995-01-01 GMT" to "1995-12-31 GMT"

```
Shannon's entropy on 8 cores with 100 shuffles.
x and y have length 8760 (0 NAs removed)
[calculate] X->Y transfer entropy
[calculate] Y->X transfer entropy
[bootstrap] 300 times
Done - Total time 25.47 seconds
Shannon Transfer Entropy Results:
---------------------------------------------------------
Direction       TE   Eff. TE  Std.Err.   p-value    sig
---------------------------------------------------------
X->Y   0.0244   0.0200   0.0007   0.0000   ***
Y->X   0.0088   0.0050   0.0008   0.0000   ***
---------------------------------------------------------
Bootstrapped TE Quantiles (300 replications):
---------------------------------------------------------
Direction     0%     25%     50%     75%    100%
---------------------------------------------------------
X->Y  0.0031  0.0045  0.0050  0.0056  0.0075
Y->X  0.0030  0.0043  0.0048  0.0053  0.0069
---------------------------------------------------------
Number of Observations: 8760
---------------------------------------------------------
p-values: < 0.001 '***', < 0.01 '**', < 0.05 '*', < 0.1 '.'
```

"1996-01-01 GMT" to "1996-12-31 GMT"

```
Shannon's entropy on 8 cores with 100 shuffles.
x and y have length 8784 (0 NAs removed)
[calculate] X->Y transfer entropy
[calculate] Y->X transfer entropy
[bootstrap] 300 times
Done - Total time 25.35 seconds
Shannon Transfer Entropy Results:
```

```
--------------------------------------------------------
Direction        TE   Eff. TE  Std.Err.   p-value    sig
--------------------------------------------------------
X->Y    0.0240    0.0197    0.0008    0.0000     ***
Y->X    0.0088    0.0049    0.0007    0.0000     ***
--------------------------------------------------------
Bootstrapped TE Quantiles (300 replications):
--------------------------------------------------------
Direction      0%     25%     50%     75%    100%
--------------------------------------------------------
X->Y  0.0034  0.0044  0.0049  0.0055  0.0069
Y->X  0.0032  0.0043  0.0047  0.0054  0.0073
--------------------------------------------------------
Number of Observations: 8784
--------------------------------------------------------
p-values: < 0.001 '***', < 0.01 '**', < 0.05 '*', < 0.1 '.'
```

"1997-01-01 GMT" to "1997-12-31 GMT"

```
Shannon's entropy on 8 cores with 100 shuffles.
x and y have length 8760 (0 NAs removed)
[calculate] X->Y transfer entropy
[calculate] Y->X transfer entropy
[bootstrap] 300 times
Done - Total time 25.18 seconds
Shannon Transfer Entropy Results:
--------------------------------------------------------
Direction        TE   Eff. TE  Std.Err.   p-value    sig
--------------------------------------------------------
X->Y    0.0105    0.0060    0.0007    0.0000     ***
Y->X    0.0054    0.0012    0.0008    0.4400
--------------------------------------------------------
Bootstrapped TE Quantiles (300 replications):
--------------------------------------------------------
Direction      0%     25%     50%     75%    100%
--------------------------------------------------------
X->Y  0.0033  0.0047  0.0052  0.0057  0.0080
Y->X  0.0030  0.0043  0.0048  0.0052  0.0072
--------------------------------------------------------
Number of Observations: 8760
--------------------------------------------------------
p-values: < 0.001 '***', < 0.01 '**', < 0.05 '*', < 0.1 '.'
```

"1998-01-01 GMT" to "1998-12-31 GMT"

```
Shannon's entropy on 8 cores with 100 shuffles.
x and y have length 8760 (0 NAs removed)
[calculate] X->Y transfer entropy
[calculate] Y->X transfer entropy
[bootstrap] 300 times
```

```
Done - Total time 25.19 seconds
Shannon Transfer Entropy Results:
---------------------------------------------------------
Direction       TE   Eff. TE  Std.Err.  p-value   sig
---------------------------------------------------------
X->Y   0.0199   0.0156   0.0008   0.0000   ***
Y->X   0.0083   0.0045   0.0007   0.0000   ***
---------------------------------------------------------
Bootstrapped TE Quantiles (300 replications):
---------------------------------------------------------
Direction     0%     25%     50%     75%    100%
---------------------------------------------------------
X->Y  0.0029  0.0044  0.0049  0.0054  0.0072
Y->X  0.0028  0.0044  0.0048  0.0054  0.0075
---------------------------------------------------------
Number of Observations: 8760
---------------------------------------------------------
p-values: < 0.001 '***', < 0.01 '**', < 0.05 '*', < 0.1 '.'
```

"1999-01-01 GMT" to "1999-12-31 GMT"

```
Shannon's entropy on 8 cores with 100 shuffles.
x and y have length 8760 (0 NAs removed)
[calculate] X->Y transfer entropy
[calculate] Y->X transfer entropy
[bootstrap] 300 times
Done - Total time 25.24 seconds
Shannon Transfer Entropy Results:
---------------------------------------------------------
Direction       TE   Eff. TE  Std.Err.  p-value   sig
---------------------------------------------------------
X->Y   0.0193   0.0151   0.0008   0.0000   ***
Y->X   0.0066   0.0028   0.0008   0.0233    *
---------------------------------------------------------
Bootstrapped TE Quantiles (300 replications):
---------------------------------------------------------
Direction     0%     25%     50%     75%    100%
---------------------------------------------------------
X->Y  0.0030  0.0044  0.0049  0.0054  0.0079
Y->X  0.0030  0.0042  0.0047  0.0052  0.0075
---------------------------------------------------------
Number of Observations: 8760
---------------------------------------------------------
p-values: < 0.001 '***', < 0.01 '**', < 0.05 '*', < 0.1 '.'
```

"2000-01-01 GMT" to "2000-12-31 GMT"

```
Shannon's entropy on 8 cores with 100 shuffles.
x and y have length 8784 (0 NAs removed)
[calculate] X->Y transfer entropy
```

[calculate] Y->X transfer entropy
[bootstrap] 300 times
Done - Total time 25.12 seconds
Shannon Transfer Entropy Results:

Direction	TE	Eff. TE	Std.Err.	p-value	sig
X->Y	0.0295	0.0255	0.0007	0.0000	***
Y->X	0.0072	0.0031	0.0008	0.0133	*

Bootstrapped TE Quantiles (300 replications):

Direction	0%	25%	50%	75%	100%
X->Y	0.0035	0.0046	0.0052	0.0057	0.0080
Y->X	0.0029	0.0041	0.0047	0.0052	0.0069

Number of Observations: 8784

p-values: < 0.001 '***', < 0.01 '**', < 0.05 '*', < 0.1 '.'

"2001-01-01 GMT" to "2001-12-31 GMT"
Shannon's entropy on 8 cores with 100 shuffles.
x and y have length 8760 (0 NAs removed)
[calculate] X->Y transfer entropy
[calculate] Y->X transfer entropy
[bootstrap] 300 times
Done - Total time 25.48 seconds
Shannon Transfer Entropy Results:

Direction	TE	Eff. TE	Std.Err.	p-value	sig
X->Y	0.0202	0.0158	0.0007	0.0000	***
Y->X	0.0090	0.0050	0.0008	0.0000	***

Bootstrapped TE Quantiles (300 replications):

Direction	0%	25%	50%	75%	100%
X->Y	0.0030	0.0044	0.0050	0.0054	0.0076
Y->X	0.0030	0.0044	0.0048	0.0054	0.0069

Number of Observations: 8760

p-values: < 0.001 '***', < 0.01 '**', < 0.05 '*', < 0.1 '.'

"2002-01-01 GMT" to "2002-12-31 GMT"
Shannon's entropy on 8 cores with 100 shuffles.

```
x and y have length 8760 (0 NAs removed)
[calculate] X->Y transfer entropy
[calculate] Y->X transfer entropy
[bootstrap] 300 times
Done - Total time 26.73 seconds
Shannon Transfer Entropy Results:
-------------------------------------------------------
Direction       TE   Eff. TE  Std.Err.   p-value    sig
-------------------------------------------------------
X->Y    0.0150    0.0106    0.0008    0.0000    ***
Y->X    0.0097    0.0058    0.0008    0.0000    ***
-------------------------------------------------------
Bootstrapped TE Quantiles (300 replications):
-------------------------------------------------------
Direction      0%     25%     50%     75%    100%
-------------------------------------------------------
X->Y  0.0033  0.0044  0.0048  0.0053  0.0075
Y->X  0.0032  0.0042  0.0047  0.0053  0.0079
-------------------------------------------------------
Number of Observations: 8760
-------------------------------------------------------
p-values: < 0.001 '***', < 0.01 '**', < 0.05 '*', < 0.1 '.'
```

"2003-01-01 GMT" to "2003-12-31 GMT"

```
Shannon's entropy on 8 cores with 100 shuffles.
x and y have length 8760 (0 NAs removed)
[calculate] X->Y transfer entropy
[calculate] Y->X transfer entropy
[bootstrap] 300 times
Done - Total time 25.62 seconds
Shannon Transfer Entropy Results:
-------------------------------------------------------
Direction       TE   Eff. TE  Std.Err.   p-value    sig
-------------------------------------------------------
X->Y    0.0104    0.0060    0.0008    0.0000    ***
Y->X    0.0122    0.0083    0.0007    0.0000    ***
-------------------------------------------------------
Bootstrapped TE Quantiles (300 replications):
-------------------------------------------------------
Direction      0%     25%     50%     75%    100%
-------------------------------------------------------
X->Y  0.0031  0.0046  0.0050  0.0055  0.0070
Y->X  0.0031  0.0043  0.0048  0.0053  0.0081
-------------------------------------------------------
Number of Observations: 8760
-------------------------------------------------------
p-values: < 0.001 '***', < 0.01 '**', < 0.05 '*', < 0.1 '.'
```

"2004-01-01 GMT" to "2004-12-31 GMT"

Shannon's entropy on 8 cores with 100 shuffles.
x and y have length 8784 (0 NAs removed)
[calculate] X->Y transfer entropy
[calculate] Y->X transfer entropy
[bootstrap] 300 times
Done - Total time 25.18 seconds
Shannon Transfer Entropy Results:

Direction	TE	Eff. TE	Std.Err.	p-value	sig
X->Y	0.0161	0.0117	0.0008	0.0000	***
Y->X	0.0120	0.0081	0.0008	0.0000	***

Bootstrapped TE Quantiles (300 replications):

Direction	0%	25%	50%	75%	100%
X->Y	0.0030	0.0045	0.0049	0.0055	0.0075
Y->X	0.0031	0.0044	0.0048	0.0053	0.0086

Number of Observations: 8784

p-values: < 0.001 '***', < 0.01 '**', < 0.05 '*', < 0.1 '.'

“2005-01-01 GMT" to "2005-12-31 GMT"

Shannon's entropy on 8 cores with 100 shuffles.
x and y have length 8760 (0 NAs removed)
[calculate] X->Y transfer entropy
[calculate] Y->X transfer entropy
[bootstrap] 300 times
Done - Total time 25.02 seconds
Shannon Transfer Entropy Results:

Direction	TE	Eff. TE	Std.Err.	p-value	sig
X->Y	0.0145	0.0101	0.0007	0.0000	***
Y->X	0.0099	0.0061	0.0008	0.0000	***

Bootstrapped TE Quantiles (300 replications):

Direction	0%	25%	50%	75%	100%
X->Y	0.0030	0.0044	0.0049	0.0054	0.0079
Y->X	0.0030	0.0043	0.0049	0.0054	0.0072

Number of Observations: 8760

p-values: < 0.001 '***', < 0.01 '**', < 0.05 '*', < 0.1 '.'

"2006-01-01 GMT" to "2006-12-31 GMT"

```
Shannon's entropy on 8 cores with 100 shuffles.
x and y have length 8760 (0 NAs removed)
[calculate] X->Y transfer entropy
[calculate] Y->X transfer entropy
[bootstrap] 300 times
Done - Total time 25.22 seconds
Shannon Transfer Entropy Results:
------------------------------------------------------------
Direction      TE    Eff. TE  Std.Err.  p-value    sig
------------------------------------------------------------
X->Y    0.0087    0.0043    0.0008    0.0000    ***
Y->X    0.0091    0.0051    0.0008    0.0000    ***
------------------------------------------------------------
Bootstrapped TE Quantiles (300 replications):
------------------------------------------------------------
Direction     0%      25%      50%      75%     100%
------------------------------------------------------------
X->Y  0.0032  0.0045  0.0050  0.0056  0.0077
Y->X  0.0026  0.0044  0.0049  0.0054  0.0073
------------------------------------------------------------
Number of Observations: 8760
------------------------------------------------------------
p-values: < 0.001 '***', < 0.01 '**', < 0.05 '*', < 0.1 '.'
```

"2007-01-01 GMT" to "2007-12-31 GMT"

```
Shannon's entropy on 8 cores with 100 shuffles.
x and y have length 8760 (0 NAs removed)
[calculate] X->Y transfer entropy
[calculate] Y->X transfer entropy
[bootstrap] 300 times
Done - Total time 25.49 seconds
Shannon Transfer Entropy Results:
------------------------------------------------------------
Direction      TE    Eff. TE  Std.Err.  p-value    sig
------------------------------------------------------------
X->Y    0.0215    0.0169    0.0008    0.0000    ***
Y->X    0.0066    0.0028    0.0008    0.0267     *
------------------------------------------------------------
Bootstrapped TE Quantiles (300 replications):
------------------------------------------------------------
Direction     0%      25%      50%      75%     100%
------------------------------------------------------------
X->Y  0.0031  0.0043  0.0048  0.0054  0.0071
Y->X  0.0030  0.0043  0.0048  0.0053  0.0070
------------------------------------------------------------
```

Number of Observations: 8760
----
p-values: < 0.001 '***', < 0.01 '**', < 0.05 '*', < 0.1 '.'

"2008-01-01 GMT" to "2008-12-31 GMT"
Shannon's entropy on 8 cores with 100 shuffles.
x and y have length 8784 (0 NAs removed)
[calculate] X->Y transfer entropy
[calculate] Y->X transfer entropy
[bootstrap] 300 times
Done - Total time 26.03 seconds
Shannon Transfer Entropy Results:

Direction	TE	Eff. TE	Std.Err.	p-value	sig
X->Y	0.0207	0.0163	0.0007	0.0000	***
Y->X	0.0113	0.0075	0.0008	0.0000	***

Bootstrapped TE Quantiles (300 replications):

Direction	0%	25%	50%	75%	100%
X->Y	0.0032	0.0045	0.0050	0.0055	0.0084
Y->X	0.0031	0.0042	0.0047	0.0052	0.0073

Number of Observations: 8784
----
p-values: < 0.001 '***', < 0.01 '**', < 0.05 '*', < 0.1 '.'

"2009-01-01 GMT" to "2009-12-31 GMT"
Shannon's entropy on 8 cores with 100 shuffles.
x and y have length 8760 (0 NAs removed)
[calculate] X->Y transfer entropy
[calculate] Y->X transfer entropy
[bootstrap] 300 times
Done - Total time 26.25 seconds
Shannon Transfer Entropy Results:

Direction	TE	Eff. TE	Std.Err.	p-value	sig
X->Y	0.0117	0.0071	0.0008	0.0000	***
Y->X	0.0090	0.0047	0.0008	0.0000	***

Bootstrapped TE Quantiles (300 replications):

Direction	0%	25%	50%	75%	100%
X->Y	0.0035	0.0045	0.0050	0.0056	0.0077

```
Y->X  0.0032  0.0044  0.0049  0.0054  0.0073
-------------------------------------------------------
Number of Observations: 8760
-------------------------------------------------------
p-values: < 0.001 '***', < 0.01 '**', < 0.05 '*', < 0.1 '.'
```

"2010-01-01 GMT" to "2010-12-31 GMT"

```
Shannon's entropy on 8 cores with 100 shuffles.
x and y have length 8760 (0 NAs removed)
[calculate] X->Y transfer entropy
[calculate] Y->X transfer entropy
[bootstrap] 300 times
Done - Total time 25.76 seconds
Shannon Transfer Entropy Results:
-------------------------------------------------------
Direction       TE   Eff. TE  Std.Err.   p-value    sig
-------------------------------------------------------
X->Y    0.0147    0.0103    0.0007    0.0000    ***
Y->X    0.0104    0.0066    0.0008    0.0000    ***
-------------------------------------------------------
Bootstrapped TE Quantiles (300 replications):
-------------------------------------------------------
Direction     0%     25%     50%     75%    100%
-------------------------------------------------------
X->Y  0.0030  0.0045  0.0049  0.0056  0.0074
Y->X  0.0031  0.0044  0.0049  0.0054  0.0072
-------------------------------------------------------
Number of Observations: 8760
-------------------------------------------------------
p-values: < 0.001 '***', < 0.01 '**', < 0.05 '*', < 0.1 '.'
```

"2011-01-01 GMT" to "2011-12-31 GMT"

```
Shannon's entropy on 8 cores with 100 shuffles.
x and y have length 8760 (0 NAs removed)
[calculate] X->Y transfer entropy
[calculate] Y->X transfer entropy
[bootstrap] 300 times
Done - Total time 24.91 seconds
Shannon Transfer Entropy Results:
-------------------------------------------------------
Direction       TE   Eff. TE  Std.Err.   p-value    sig
-------------------------------------------------------
X->Y    0.0122    0.0076    0.0008    0.0000    ***
Y->X    0.0078    0.0041    0.0007    0.0000    ***
-------------------------------------------------------
Bootstrapped TE Quantiles (300 replications):
-------------------------------------------------------
Direction     0%     25%     50%     75%    100%
```

```
--------------------------------------------------------
X->Y  0.0030  0.0045  0.0050  0.0054  0.0071
Y->X  0.0031  0.0045  0.0049  0.0055  0.0074
--------------------------------------------------------
Number of Observations: 8760
--------------------------------------------------------
p-values: < 0.001 '***', < 0.01 '**', < 0.05 '*', < 0.1 '.'
```

"2012-01-01 GMT" to "2012-12-31 GMT"

```
Shannon's entropy on 8 cores with 100 shuffles.
x and y have length 8784 (0 NAs removed)
[calculate] X->Y transfer entropy
[calculate] Y->X transfer entropy
[bootstrap] 300 times
Done - Total time 25.66 seconds
Shannon Transfer Entropy Results:
--------------------------------------------------------
Direction       TE   Eff. TE  Std.Err.   p-value    sig
--------------------------------------------------------
X->Y    0.0114    0.0070    0.0008    0.0000    ***
Y->X    0.0103    0.0064    0.0007    0.0000    ***
--------------------------------------------------------
Bootstrapped TE Quantiles (300 replications):
--------------------------------------------------------
Direction     0%     25%     50%     75%    100%
--------------------------------------------------------
X->Y  0.0032  0.0044  0.0049  0.0054  0.0074
Y->X  0.0031  0.0043  0.0048  0.0054  0.0074
--------------------------------------------------------
Number of Observations: 8784
--------------------------------------------------------
p-values: < 0.001 '***', < 0.01 '**', < 0.05 '*', < 0.1 '.'
```

"2013-01-01 GMT" to "2013-12-31 GMT"

```
Shannon's entropy on 8 cores with 100 shuffles.
x and y have length 8760 (0 NAs removed)
[calculate] X->Y transfer entropy
[calculate] Y->X transfer entropy
[bootstrap] 300 times
Done - Total time 24.85 seconds
Shannon Transfer Entropy Results:
--------------------------------------------------------
Direction       TE   Eff. TE  Std.Err.   p-value    sig
--------------------------------------------------------
X->Y    0.0130    0.0085    0.0008    0.0000    ***
Y->X    0.0073    0.0031    0.0008    0.0100     *
--------------------------------------------------------
Bootstrapped TE Quantiles (300 replications):
```

```
---------------------------------------------------------
Direction     0%     25%     50%     75%    100%
---------------------------------------------------------
X->Y  0.0031  0.0046  0.0051  0.0057  0.0081
Y->X  0.0032  0.0043  0.0048  0.0053  0.0071
---------------------------------------------------------
Number of Observations: 8760
---------------------------------------------------------
p-values: < 0.001 '***', < 0.01 '**', < 0.05 '*', < 0.1 '.'
```

"2014-01-01 GMT" to "2014-12-31 GMT"

```
Shannon's entropy on 8 cores with 100 shuffles.
x and y have length 8760 (0 NAs removed)
[calculate] X->Y transfer entropy
[calculate] Y->X transfer entropy
[bootstrap] 300 times
Done - Total time 24.87 seconds
Shannon Transfer Entropy Results:
---------------------------------------------------------
Direction       TE   Eff. TE  Std.Err.  p-value    sig
---------------------------------------------------------
X->Y    0.0172    0.0129    0.0007    0.0000    ***
Y->X    0.0094    0.0055    0.0007    0.0000    ***
---------------------------------------------------------
Bootstrapped TE Quantiles (300 replications):
---------------------------------------------------------
Direction     0%     25%     50%     75%    100%
---------------------------------------------------------
X->Y  0.0032  0.0044  0.0048  0.0053  0.0076
Y->X  0.0029  0.0043  0.0048  0.0053  0.0072
---------------------------------------------------------
Number of Observations: 8760
---------------------------------------------------------
p-values: < 0.001 '***', < 0.01 '**', < 0.05 '*', < 0.1 '.'
```

"2015-01-01 GMT" to "2015-12-31 GMT"

```
Shannon's entropy on 8 cores with 100 shuffles.
x and y have length 8760 (0 NAs removed)
[calculate] X->Y transfer entropy
[calculate] Y->X transfer entropy
[bootstrap] 300 times
Done - Total time 24.91 seconds
Shannon Transfer Entropy Results:
---------------------------------------------------------
Direction       TE   Eff. TE  Std.Err.  p-value    sig
---------------------------------------------------------
X->Y    0.0088    0.0043    0.0007    0.0000    ***
Y->X    0.0065    0.0030    0.0008    0.0100     *
```

Bootstrapped TE Quantiles (300 replications):

Direction	0%	25%	50%	75%	100%
X->Y	0.0031	0.0043	0.0048	0.0053	0.0078
Y->X	0.0029	0.0043	0.0048	0.0053	0.0071

Number of Observations: 8760
----
p-values: < 0.001 '***', < 0.01 '**', < 0.05 '*', < 0.1 '.'

"2016-01-01 GMT" to "2016-12-31 GMT"

Shannon's entropy on 8 cores with 100 shuffles.
x and y have length 8784 (0 NAs removed)
[calculate] X->Y transfer entropy
[calculate] Y->X transfer entropy
[bootstrap] 300 times
Done - Total time 25.46 seconds
Shannon Transfer Entropy Results:

Direction	TE	Eff. TE	Std.Err.	p-value	sig
X->Y	0.0184	0.0140	0.0008	0.0000	***
Y->X	0.0084	0.0047	0.0008	0.0000	***

Bootstrapped TE Quantiles (300 replications):

Direction	0%	25%	50%	75%	100%
X->Y	0.0032	0.0043	0.0048	0.0054	0.0074
Y->X	0.0032	0.0044	0.0049	0.0055	0.0074

Number of Observations: 8784
----
p-values: < 0.001 '***', < 0.01 '**', < 0.05 '*', < 0.1 '.'

"2017-01-01 GMT" to "2017-12-31 GMT"

Shannon's entropy on 8 cores with 100 shuffles.
x and y have length 8760 (0 NAs removed)
[calculate] X->Y transfer entropy
[calculate] Y->X transfer entropy
[bootstrap] 300 times
Done - Total time 24.96 seconds
Shannon Transfer Entropy Results:

Direction TE Eff. TE Std.Err. p-value sig

X->Y	0.0170	0.0125	0.0008	0.0000	***
Y->X	0.0083	0.0044	0.0008	0.0000	***

Bootstrapped TE Quantiles (300 replications):

Direction	0%	25%	50%	75%	100%
X->Y	0.0034	0.0045	0.0050	0.0056	0.0076
Y->X	0.0031	0.0043	0.0048	0.0053	0.0074

Number of Observations: 8760

----

p-values: < 0.001 '***', < 0.01 '**', < 0.05 '*', < 0.1 '.'
